# Impact of Bedside Re-Explorations in a Cardiovascular Surgery Intensive Care Unit Led by Surgeons †

**DOI:** 10.3390/jcm10194288

**Published:** 2021-09-22

**Authors:** Alessandro Affronti, Elena Sandoval, Anna Muro, Jose Hernández-Campo, Eduard Quintana, Daniel Pereda, Jorge Alcocer, Robert Pruna-Guillen, Manuel Castellà

**Affiliations:** Cardiovascular Surgery, Hospital Clínic, University of Barcelona, c/Villarroel 170 Esc 1 5th Floor, 08036 Barcelona, Spain; affronti@clinic.cat (A.A.); amuro@clinic.cat (A.M.); jahernandez@clinic.cat (J.H.-C.); equintan@clinic.cat (E.Q.); dpereda@clinic.cat (D.P.); alcocer@clinic.cat (J.A.); pruna@clinic.cat (R.P.-G.); mcaste@clinic.cat (M.C.)

**Keywords:** postoperative management, intensive care unit, surgical re-explorations, mediastinitis

## Abstract

Surgical re-explorations represent 3–5% of all cardiac surgery. Concerns regarding mortality and major morbidity of re-explorations in the intensive care unit (ICU) setting exist. We sought to investigate whether they may have different outcomes compared with those performed in the operating room (OR). Single center retrospective review of patients who underwent mediastinal re-exploration in the ICU or in the OR after cardiac surgery. Mediastinal re-explorations were also classified as: “planned” and “unplanned”. Primary outcome was 30-day mortality, secondary outcomes include deep sternal wound infection (DSWI), sepsis, ICU and hospital length of stay, prolonged intubation (>72 h), tracheostomy, pneumonia, acute kidney injury requiring dialysis and stroke. Between 2010 and 2019, 195 of 7263 patients (2.7%) underwent mediastinal re-exploration after cardiac surgery. More patients in the ICU group experienced two or more re-explorations (30.3% vs. 2.3%, *p* < 0.001), a higher incidence of postoperative pneumonia (22% vs. 7%, *p* = 0.004), prolonged intubation (46.8% vs. 19.8%, *p* < 0.001) and longer hospital stay (30.3 ± 34.2 vs. 20.8 ± 18.3 days, *p* = 0.014). There were no differences in mortality between ICU and OR (16.5% vs. 13.9%, *p* = 0.24) nor in sepsis (14.7% vs. 7%, *p* = 0.91) and DSWI rates (1.8% vs. 1.2%, *p* = 0.14). Re-explorations in the ICU were not associated with increased mortality, sepsis and mediastinitis rate.

## 1. Introduction

Mediastinal re-explorations in the immediate postoperative period account for 3–5% of all cardiac surgical procedures [1,2,3,4]. The most frequent emergent causes are excessive postoperative bleeding, cardiac arrest, arrhythmias and cardiac tamponade. However, some patients cannot undergo sternal closure at the end of the index procedure because of hemodynamic instability, myocardial edema or persistent bleeding, despite accurate hemostasis and correction of any coagulation derangement. In such a case, the mediastinum is packed with sponges and the sternum is left open to be closed after the underlying condition has been solved.

There is limited literature on the safety of re-explorations carried out in the intensive care unit (ICU). Potential advantages include avoiding transportation of unstable patients and any delay due to unavailable operating room (OR). On the other hand, concerns regarding mortality and major morbidity still remain [5,6,7,8].

The aim of this study was to evaluate the clinical outcomes of surgical re-explorations performed in the ICU compared with those performed in the OR. We also sought to investigate whether re-explorations performed in the ICU in the setting of an emergent situation such as cardiac arrest, cardiac tamponade or unexpected massive bleeding (“unplanned”) had different outcomes in comparison to those performed electively, as in case of delayed sternal closure (“planned”).

## 2. Materials and Methods

### 2.1. Ethics

The local Ethics Committee/Institutional Review Board approved the use of anonymized patient data, which waived the need for individual patient consent (HCB/2020/0625; 11 June 2020).

### 2.2. Study Design

This is a single-center retrospective review of patients who underwent cardiac surgery and subsequently required mediastinal re-exploration in the immediate postoperative period at our institution.

### 2.3. Group Assignment

Patients were divided into two groups depending on where they underwent surgical revision, either in the cardiovascular ICU or in the OR.

### 2.4. Exclusion Criteria

Patients whose index operation was heart transplantation were excluded from the study because the immunosuppressive therapy associated with surgical re-exploration(s) could have been an important bias in determining the infection rate. Similarly, patients who needed mechanical circulatory support in the postoperative period were not included due to the potential need for multiple re-explorations.

### 2.5. Classification of Re-Explorations

Some patients underwent more than one procedure. In that case, they were classified according to the location of the first re-exploration (ICU or OR).

Re-explorations were also classified as:

- Unplanned, due to massive bleeding, cardiac tamponade or cardiac arrest;
- Planned, when patients were intentionally left with the chest open because of persistent general bleeding, hemodynamic instability or myocardial edema and underwent delayed sternal closure (DSC).

In order to avoid any possible bias, planned re-explorations were also analyzed according to the site where the procedure was performed (OR vs. ICU).

### 2.6. Definitions

Definitions of preoperative and intraoperative variables as well as postoperative outcomes are according to the STS adult cardiac surgery database collection [9].

Mortality at 30 days after surgery was defined as any death occurring within the first 30 days after the index operation (either after discharge or in-hospital) [9]. Inter-hospital transfer was not considered discharge.

We used the Centers for Disease Control and Prevention’s (CDC) definition of DSWI. In particular, a DSWI requires positive culture results of surgical sites or drainage from the mediastinal area or evidence of infection during surgical re-exploration or fever, sternal instability and positive blood culture results [10].

The universal definition of perioperative bleeding in adult cardiac surgery was used to define surgical bleeding and massive bleeding [11]. Non-surgical bleeding was defined as an excessive blood loss from the chest tubes, without a specific surgical cause was found at mediastinal re-exploration and generally secondary to coagulation derangement.

Preoperative risk stratification was performed using the Euroscore II [12].

### 2.7. Outcomes of Interest

The primary outcome was mortality at 30 days after surgery (either after discharge or in-hospital), Secondary outcomes included ICU and hospital length of stay, prolonged intubation (>72 h), tracheostomy, pneumonia, DSWI, sepsis, AKI requiring dialysis and stroke.

### 2.8. Data Collection

Demographics as well as preoperative, intraoperative and postoperative data were extracted from the institutional database and supplemented by a detailed review of medical records.

### 2.9. Technique for Re-Exploration in the ICU

The cardiovascular ICU has 16 beds, distributed in double or single rooms. All are equipped with a positive pressure ventilation system which minimizes recirculation of air so that any airborne particle originating in the room can be filtered out [13].

The unit is under the responsibility of the surgeon on duty, fully trained in cardiac surgery critical care (intubation, mechanical ventilation and pharmacological management, central lines and Swan-Ganz catheter insertion, chest tube and intra-aortic balloon pump insertion, etc.), with two staff surgeons available.

The decision to perform re-exploration and whether to do it in the ICU or in the OR, does not come from a specific protocol or flowchart, but is based on a global evaluation of the patient stability and the clinical setting after optimizing any underlying coagulopathy, in case of bleeding. It is normally undertaken by the surgeon responsible for the patient with the assistance of the surgeon on call.

The ICU nursing staff are specially trained in assisting in reoperations, all of them are CALS (Cardiac Surgery Advanced Life Support) certified according to the STS/EACTS guidelines and retrained on an annual basis [14,15,16]. For this reason, anesthesia and scrub nurse support is not routinely necessary during ICU re-explorations.

The operating site is cleansed with a chlorhexidine solution, prepped and draped similarly to standard operations in the OR. Two sets of sterile surgical instruments are always available in the ICU, as well as an emergent opening set. Access to the room is restricted to those directly involved in the bedside re-exploration: normally, a surgeon and an assistant (another surgeon or an ICU nurse) and one or two circulating nurses for external assistance, managing of mechanical ventilation and medication. Antibiotic prophylaxis is based on re-dosing the one administered in the OR. If the chest is left opened, antibiotics are maintained until sternal closure.

In the case of delayed sternal closure, the mediastinum is usually packed with sponges and sterile cotton wool. A rubber patch obtained from an Esmarch bandage is then applied and stapled to the skin, followed by a Steri-drape™ (3M, St. Paul, MN, USA) membrane. This technique aims at preserving asepsis and maintains a negative pressure for adequate drainage of the cavity. A visual advantage of the elastic patch is that it retracts into the mediastinum when negative pressure is applied but tends to become convex if negative pressure is lost or if clots accumulate in the mediastinum [17].

The definitive sternal closure is performed as per routine after removal of packing, accurate inspection of all surgical sites and irrigation of the pericardium with diluted iodine solution.

### 2.10. Statistical Analysis

Continuous variables were expressed as the mean ± standard deviation (SD) or median and interquartile range, as appropriate, and were evaluated by Student’s *t*-test; categorical data were expressed as percentages and were evaluated using χ^2^ or Fisher’s exact test. A significance level of 5% was set for all the analyses. All statistical analyses were performed using R freeware version 4.0.3 (R foundation for Statistical Computing, Vienna, Austria) [18].

## 3. Results

Between January 2010 and December 2019, 195 (2.7%) of 7263 patients who underwent cardiac surgery at our institution required surgical re-exploration(s) in the immediate postoperative period. Of these, 86 (44.1%) were performed in the OR and the remaining 109 (55.9%) were performed in the ICU.

**Figure 1:**
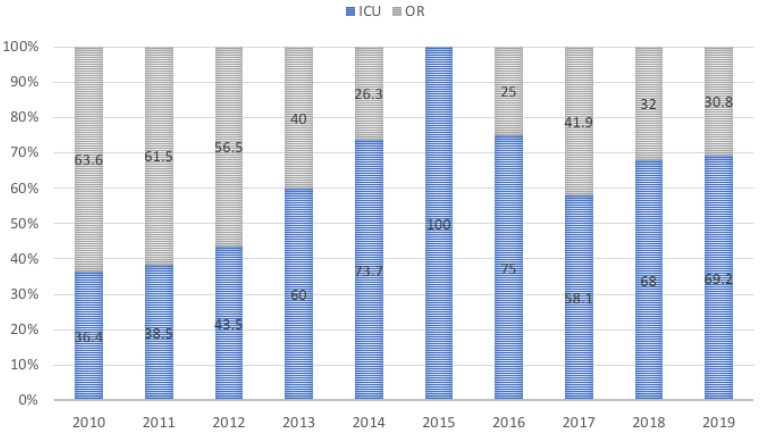
Proportion of the annual ICU/OR re-explorations over the years. ICU: Intensive Care Unit, OR: Operating Room.

The proportion of re-explorations in the ICU versus OR per year is shown in Figure 1.

A prevalence of the former over the latter was observed over the study period. This trend became more evident in the last years mainly due to the increasing confidence of the ICU staff in carrying out this procedure, along with its perceived advantages such as avoiding transportation of unstable patients or any delay due to OR set-up.

As summarized in Table 1, the two cohorts of patients (ICU and OR) were homogeneous in terms of preoperative characteristics, with the exception of age and serum creatinine levels, which were both higher in the ICU group. The surgical profile of the two groups was also comparable with regards to the index procedures performed and risk stratification, as corroborated by the similar Euroscore II. However, the ICU group had a significantly higher proportion of patients who underwent primary surgery under urgent/emergent conditions and, conversely, elective index operations were more frequent in the OR group.

Index procedures performed encompass the entire spectrum of adult cardiac surgery, including many complex operations such as multiple valve surgery, combined surgery and reinterventions, as demonstrated by the high average Euroscore II (Table 2).

Table 3 summarizes the re-exploration details, including the time interval from the index cardiac surgical procedure and the causes that led to re-exploration. Despite not statistically significant, 46.8% of the ICU re-explorations were performed in the first 24 h, whereas only 38% of those performed in the OR re-explorations were carried out in the same timeframe. The main reason for re-exploration in both groups was bleeding, followed by cardiac tamponade. Cardiac arrest as a cause for re-exploration was significantly higher in the ICU group. More patients in the ICU group required more than one re-exploration.

Outcomes are presented in Table 4; there were no differences in 30-day mortality. The only difference in terms of cause of mortality between groups is massive bleeding which resulted significantly higher in the ICU cohort, however the absolute figures (1 case in the OR group vs. 2 cases in the ICU group) are too small to draw any conclusion (Table 5).

The ICU group had a longer hospital length of stay as well as higher incidence of prolonged ventilation and postoperative pneumonia. There were no differences in the other secondary outcomes.

Table 6 reports the outcomes of planned re-explorations according to the site where the procedure took place (OR vs. ICU).

Table 7 and Table 8 specifically depict the results of surgical re-explorations in the ICU, where, of a total of 109 cases, 84 (77.1%) were “unplanned” and 25 (22.9%) were “planned.” No differences in pre-operative variables were noted between the two cohorts, except for a significantly higher proportion of patients in the “planned” group who underwent primary surgery under urgent/emergent conditions. This group also showed longer CPB and ischemic times as well as a higher proportion of patients who underwent hypothermic circulatory arrest. There were no differences in outcomes, except for higher 30-day mortality (32% vs. 11.9%, *p* = 0.029 and prolonged ventilation in the “planned” group (68% vs. 40.5%, *p* = 0.022).

## 4. Discussions

In our study, 2.7% of the overall population required surgical re-exploration after cardiac surgery, which is consistent with the existing literature, showing re-exploration rates between 1–4% [1,2,3,4,8].

The main findings of our study are that re-explorations in the ICU setting were associated with similar mortality and ICU length of stay to those carried out in the OR. These results are consistent with those previously reported by Fiser et al. [19], Charalambous et al. [20] and Kaiser et al. [21].

The time interval between the primary cardiac surgical procedure and the re-exploration is not different between the two cohorts (same median: 1 day). The presence of a 24-h surgeon can be an extra asset to shorten the time interval from decision-making to performance and decide where to do it depending on the clinical situation of each patient.

Re-explorations in the ICU resulted in longer hospital length of stay, longer intubation times and higher incidence of postoperative pneumonia. A possible explanation may be the higher proportion of patients who underwent urgent/emergent index surgery in this group and the fact that those patients needed more re-explorations.

These operations may have been performed under anticoagulant/antiplatelet treatment not stopped in due time, or in worse underlying situation. This may have contributed to a higher need of more than one re-exploration, which could justify the longer intubation times and hospital length of stay. It is well known that longer intubation time increases the risk of ventilator-associated pneumonia [22,23]. Older age and higher preoperative serum creatinine in this group may have also played a role, but further studies are needed to confirm this.

Lastly, it must be considered also that in the ICU group a higher proportion of patients underwent emergent re-exploration for cardiac arrest compared to those in the OR group.

As pointed out, 33 patients (30.3%) in the ICU group needed more than one re-exploration versus only 2 patients (2.3%) in the OR group. Regarding the number of re-explorations, the 2 groups show a similar range and the same median. The causes that led to multiple re-explorations in the ICU group include persistent bleeding, delayed sternal closure and cardiac arrest/refractory arrythmia. In the OR cohort both cases were secondary to persistent bleeding. This difference in multiple re-explorations rate may be multifactorial. As discussed above, it is likely that the profile of the patients undergone re-exploration in the ICU setting was at increased risk (more patients underwent emergent/urgent index procedure, 6.4% of patients underwent the first re-exploration for cardiac arrest). Apart from this, another aspect must be taken into account: the typical case in this group is a patient who underwent a complex surgery, subsequently underwent a first re-exploration in the ICU for bleeding and was left with the chest opened. Then, underwent delayed sternal closure once the conditions permitted. This DSC accounts for “second re-exploration”. The advantage of performing a DSC in the ICU is that it can be planned anytime without the need of extra staff and without altering the planned surgical activity.

Due to the limited number or multiple re-explorations in the OR group (only 2 cases) it was impossible to perform any further subanalysis statistically meaningful.

The study showed no difference regarding other postoperative complications such as stroke, AKI requiring dialysis and tracheostomy.

Sepsis and DSWI rates were also similar to those of re-explorations performed in the OR. This has been previously demonstrated by other authors [19,24] and is particularly important since it corroborates our hypothesis that re-explorations could be safely carried out in an ICU environment without an increase in mortality and infection rate when performed in accordance with good practice rules, particularly concerning asepsis.

The subanalysis of “planned” re-explorations according to the site where the procedure was performed (OR vs. ICU) showed no differences in terms of mortality or other outcomes, in particular sepsis and DSWI. The only exception was the percentage of patients who needed intubation > 72 h, which resulted significantly higher in the ICU cohort. This data is consistent with the fact that DSC in the ICU is typically the last procedure in of a series of multiple re-explorations, where bleeding/tamponade/cardiac arrest is normally the first event, thus a longer intubation time is the logical consequence.

When we specifically focus on the subgroup of re-explorations in the ICU, the main finding is that unplanned procedures had lower mortality rates than planned re-explorations, which points to an acute and effective resolution of emergent life-threatening conditions when surgery is performed without delay. Indeed, a frequent scenario in this subgroup was the case of a patient being quickly re-operated in the ICU because of massive bleeding, cardiac arrest or cardiac tamponade, who then experienced a normal postoperative course once the acute condition was fixed.

Conversely, the higher mortality of “planned” procedures is likely due to worse clinical conditions at the time of initial surgery, as suggested by the higher Euroscore II in this group as well as the greater proportion of patient who were initially operated in urgent/emergent condition, the longer CPB and cardiac ischemic time and the higher percentage of patients who needed deep hypothermic circulatory arrest. These “planned” operations were delayed chest closure in most cases. These usually take place once coagulopathy is fixed and hemodynamics are stable, normally in postoperative day one or two. This timeframe may also be the reason for the higher proportion of patients who experienced prolonged intubation and hospital length of stay.

It is important to point out, that despite seeming two different situations (a life threatening one versus a delayed chest closure) the rate of sepsis and DSWI is not different, proving the re-explorations in the ICU are safe even in emergent situations.

### Limitations

This study is limited by the relatively small sample size, its retrospective design and the fact that it represents a single-center experience with a very specific organization, in particular the continuous presence of a cardiac surgeon on duty and the nurse staff trained in re-explorations and CALS certified, thus we do not know to what extent our results could be applied on a broader scale.

## 5. Conclusions

In our study, reinterventions performed in the ICU were not associated with increased 30-day mortality, mediastinal infections or sepsis, despite the need for repeated explorations, a higher incidence of postoperative pneumonia, prolonged intubation and longer hospital stay. Other potential advantages include avoiding transportation of unstable patients and the fact that planned surgical activity for the day after is not affected since ICU intervention does not require the use of an operating theater and OR staff.

Our study was not designed to detect any cost-effectiveness benefit; however, a possible advantage in favor of re-explorations in the ICU in these terms has been demonstrated by other authors [21,25].

A dedicated cardiovascular surgery ICU with trained personnel, appropriate infrastructure (rooms with positive pressure ventilation systems, adequate surgical material readily available, etc.) is mandatory to achieve good results.

## Figures and Tables

**Table 1:** Baseline characteristics and Intraoperative details.

	OR *n* = 86	ICU *n* = 109	*p* Value
Age (y)	64.4 ± 13.1	68.7 ± 9.8	0.005
Female	31 (36%)	38 (34.9%)	0.28
BMI (Kg/m^2^)	27.1 ± 4.7	26.7 ± 4.3	0.29
Active smoker	6 (7%)	16 (14.7%)	0.77
Diabetes	17 (19.8%)	27 (24.8%)	0.41
Hypertension	54 (62.8%)	82 (75.2%)	0.06
COPD	15 (17.4%)	13 (11.9%)	0.27
Serum creatinine (mg/dL)	1.1 ± 0.5	1.3 ± 1.2	0.034
LVEF (%)	53.6 ± 12.6	54.5 ± 12.5	0.33
Pre-op Anticoagulant	31 (36%)	25 (22.9%)	0.64
Pre-op Antiplatelets other than ASA	6 (7%)	14 (12.8%)	0.18
Active Endocarditis	3 (3.5%)	9 (8.2%)	0.28
Euroscore II	6.8 ± 9.8	9.6 ± 13	0.5
Status of Index procedure:			
elective	68 (79.1%)	66 (60.5%)	0.006
urgent/emergent	18 (20.9%)	43 (39.5%)	0.006
CPB (mins)	144.8 ± 78.8	153.8 ± 88.2	0.23
Cardiac ischemic time (mins)	96.3 ± 48.8	97.2 ± 45.4	0.45
Circulatory arrest (mins)	57.1 ± 50.9	44.5 ± 42.1	0.24
Deep hypothermia	11 (12.8%)	23 (21.1%)	0.13
Previous cardiac surgery	12 (13.9%)	22 (20.2%)	0.25

ASA: Acetyl Salicylic Acid, CPB: cardiopulmonary bypass, BMI: body mass index, COPD: chronic obstructive pulmonary disease, ICU: intensive care unit, LVEF: left ventricular ejection fraction, OR: operating room.

**Table 2:** Index procedures.

Index Procedure	OR *n* = 86	ICU *n* = 109
CABG	12 (13.9%)	18 (16.5%)
AVR	13 (15.1%)	13 (11.9%)
AVR + MVR	3 (3.5%)	4 (3.7%)
AVR + MVR + TA	3 (3.5%)	3 (2.7%)
AVR + MVrep	-	2 (1.8%)
AVR + AA	3 (3.5%)	3 (2.7%)
AVR + CABG	2 (2.3%)	3 (2.7%)
AVR + AA + CABG	2 (2.3%)	-
AVR + AA + CABG + Maze	1 (1.2%)	-
AVR + AA + MVrep	-	1 (0.9%)
AVR + AA + MVrep + CABG	-	1 (0.9%)
AVR + TA	2 (2.3%)	-
MVR	3 (3.5%)	5 (4.6%)
MVrep	3 (3.5%)	2 (1.8%)
AA replacement	3 (3.5%)	2 (1.8%)
MVR + TA	4 (4.6%)	3 (2.7%)
MVR + TA + CABG	-	1 (0.9%)
MVR + TVR	-	1 (0.9%)
MVR +CABG	-	4 (3.7%)
MVR + CABG + AA	-	1 (0.9%)
MVrep + TA	1 (1.2%)	1 (0.9%)
MVrep + CABG	2 (2.3%)	2 (1.8%)
MVrep +Maze + PFO	1 (1.2%)	-
MVR + AT + Maze	3 (3.5%)	-
CABG + Maze	1 (1.2%)	-
Bentall operation	3 (3.5%)	6 (5.5%)
Bentall operation open distal	-	5 (4.6%)
Bentall operation homograft	-	2 (1.8%)
Bentall operation + Arch replacement	6 (7%)	10 (9.2%)
Bentall operation + MVR	1 (1.2%)	-
Bentall operation + CABG	-	1 (0.9%)
Commando operation	3 (3.5%)	2 (1.8%)
Commando operation + TA + CABG	-	1 (0.9%)
Elephant trunk	4 (4.6%)	4 (3.7%)
FET	1 (1.2%)	2 (1.8%)
David operation + MVrep	1 (1.2%)	-
AVrep + MVR + TA	1 (1.2%)	-
AVrep + AA + Maze	1 (1.2%)	-
Pericardiectomy	1 (1.2%)	-
Thoraco-abdominal aneurysm	1 (1.2%)	1 (0.9%)
PPVV ablation	1 (1.2%)	-
Infarct exclusion	-	1 (0.9%)
Septal Myectomy	-	1 (0.9%)
Septal Myectomy + AVR + MVR + TA	-	1 (0.9%)
LV Aneurysm repair + CABG	-	1 (0.9%)
PEA	-	1 (0.9%)

AA: ascending aorta replacement, ASD: atrial septal defect, AVR: aortic valve replacement, AVrep: aortic valve repair, CABG: coronary aortic bypass grafting, FET: frozen elephant trunk, LV: left ventricle, MVR: mitral valve replacement, MVrep: mitral valve repair, PFO: patent foramen ovale, PEA: pulmonary endoarterectomy, PPVV: pulmonary veins, TA: tricuspid annuloplasty, TVR: tricuspid valve replacement.

**Table 3:** Re-exploration(s) details.

	OR *n* = 86	ICU *n* = 109	*p* Value
Time from surgery (d):			
<24 h	33 (38.4%)	51 (46.8%)	0.24
24–48 h	6 (7%)	12 (11%)	0.33
Median; range (d)	1; 0–14	1; 0–13	
Unplanned	67 (77.9%)	84 (77.1%)	0.89
Planned	19 (22.1%)	25 (22.9%)	0.89
Reason for re-exploration:			
Bleeding	44 (51.2%)	47 (43.1%)	0.26
Cardiac tamponade	23 (26.7%)	28 (25.7%)	0.56
Cardiac arrest	-	7 (6.4%)	0.018
Delayed sternal closure	19 (22.1%)	25 (22.9%)	0.89
Other	-	2 (1.8%)	0.5
Non-surgical bleeding	31/67 (46.3%)	38/74 (51.3%)	0.95
2 or more re-explorations	2 (2.3%)	33 (30.3%)	<0.001
Multiple re-explorations: details			
range (median)	2-4 (2)	2-3 (2)	
Cause of multiple re-explorations:			
Persistent bleeding	2 (100%)	13 (39.4%)	
Delayed sternal closure	-	14 (42.4%)	
Cardiac arrest/ refractory arrythmia	-	6 (18.2%)	

ICU: intensive care unit, OR: operating room.

**Table 4:** Outcomes.

	OR *n* = 86	ICU *n* = 109	*p* Value
30-day mortality	12 (13.9%)	18 (16.5%)	0.24
ICU stay (d)	14.1 ± 19.8	19.3 ± 29.9	0.89
Hospital stay (d)	20.8 ± 18.3	30.3 ± 34.2	0.014
Intubation > 72 h	17 (19.8%)	51 (46.8%)	<0.001
Pneumonia	6 (7%)	24 (22%)	0.004
Tracheostomy	9 (10.5%)	14 (12.8%)	0.61
AKI requiring dialysis	13 (15.1%)	25 (22.9%)	0.17
Sepsis	6 (7%)	16 (14.7%)	0.91
DSWI	1 (1.2%)	2 (1.8%)	0.14
Stroke	3 (3.5%)	5 (2.7%)	0.70

AKI: acute kidney injury, DSWI: deep sternal wound infection; ICU: intensive care unit, OR: operating room.

**Table 5:** Cause of death.

	OR *n* = 12	ICU *n* = 18	*p* Value
MOF	7 (58.3%)	13 (72.2%)	0.33
massive bleeding	1 (8.3%)	2 (11.1%)	0.02
neurologic	2 (16.7%)	1 (5.6%)	0.32
cardiac	2 (16.7%)	2 (11.1%)	0.66

MOF: multiple organ failure, ICU: intensive care unit, OR: operating room.

**Table 6:** Outcomes of planned re-explorations analyzed by OR versus ICU.

	OR *n* = 19	ICU *n* = 25	*p* Value
30-day mortality	2 (10.9%)	9 (36%)	0.15
Intubation > 72 h	6 (31.6%)	17 (68%)	0.03
Pneumonia	3 (15.8%)	7 (28%)	0.47
Tracheostomy	6 (31.6%)	6 (24%)	0.73
AKI requiring dialysis	3 (15.8%)	9 (36%)	0.31
Sepsis	5 (26.3%)	5 (20%)	0.39
DSWI	1 (5.3%)	-	0.43
Stroke	2 (10.9%)	1 (4%)	0.56

AKI: acute kidney injury, DSWI: deep sternal wound infection, ICU: intensive care unit, OR: operating room.

**Table 7:** Re-explorations in the ICU setting: pre-op and intraoperative details.

	Planned *n* = 25	Unplanned *n* = 84	*p* Value
Age	65.4 ± 12.4	69.6 ± 8.5	0.27
Female	12 (48%)	26 (30.9%)	0.12
BMI	38.2 ± 23.8	26.9 ± 4.4	0.13
Active smoker	9 (36%)	45 (53.6%)	0.12
Diabetes	3 (12%)	24 (28.6%)	0.09
Hypertension	20 (80%)	62 (73.8%)	0.53
COPD	4 (16%)	10 (11.9%)	0.49
Serum creatinine (mg/dL)	1.3 ± 0.8	1.3 ± 1.3	0.49
Status of Index procedure:			
elective	5 (20%)	60 (71.4%)	<0.001
urgent/emergent	20 (80%)	24 (28.6%)	<0.001
CPB (mins)	228.8 ± 89.1	131.2 ± 73.5	<0.001
Cardiac ischemic time (mins)	129.8 ± 46.7	87.3 ± 39.5	<0.001
Circulatory arrest	13 (52%)	7 (8.3%)	<0.001
Deep hypothermia	13 (52%)	7 (8.3%)	<0.001
Previous cardiac surgery	8 (32%)	14 (16.6%)	0.93
Euroscore II	14.9 ± 18.4	8 ± 10.3	<0.05

ASA: Acetyl Salicylic Acid, BMI: body mass index, COPD: chronic obstructive pulmonary disease, ICU: intensive care unit, OR: operating room.

**Table 8:** Re-explorations in the ICU setting: Outcomes.

	Planned *n* = 25	Unplanned *n* = 84	*p* Value
30-day mortality	8 (32%)	10 (11.9%)	0.029
ICU stay (d)	26.4 ± 29.5	17.1 ± 29.7	0.88
Hospital stay (d)	36.8 ± 30	28.5 ± 35.2	0.15
Intubation > 72 h	17 (68%)	34 (40.5%)	0.022
Pneumonia	7 (28%)	17 (20.2%)	0.41
Tracheostomy	6 (24%)	8 (9.5%)	0.57
AKI requiring dialysis	9 (36%)	16 (19%)	0.76
Sepsis	5 (20%)	11 (13.1%)	0.39
DSWI	1 (4%)	1 (1.2%)	0.36
Stroke	1 (4%)	4 (4.8%)	0.87

AKI: acute kidney injury, DSWI: deep sternal wound infection, ICU: intensive care unit, OR: operating room.

## Data Availability

Data supporting reported results can be provided on request.

## Abbreviations

OR	Operating room
ICU	Intensive care unit
DSC	Delayed sternal closure
CDC	Centers for Disease Control and Prevention
DSWI	Deep sternal wound infection
AKI	Acute kidney injury
CALS	Cardiac Surgery Advanced Life Support
STS	Society for Thoracic Surgery
EACTS	European Society for Cardiothoracic Surgery
CPB	Cardiopulmonary bypass
